# Supplementary material for: The transcription-repair coupling factor Mfd associates with RNA polymerase in the absence of exogenous damage
Source: Nat Commun. 2018 Apr 20;9:1570. doi: 10.1038/s41467-018-03790-z (PMC5910403; doi:10.1038/s41467-018-03790-z)
Supplement: Supplementary file 1 — Supplementary Information [file 41467_2018_3790_MOESM1_ESM.docx]

Supplementary Information for

The transcription-repair coupling factor Mfd associates with RNA polymerase in the absence of exogenous damage

by

Han N. Ho, Antoine M. van Oijen, Harshad Ghodke

Correspondence to:

harshad@uow.edu.au

**This PDF file includes:**

Supplementary Figures 1 to 8

Supplementary Tables 1 to 7

Supplementary Notes 1 to 4

Supplementary References

Contents

[Supplementary Figures 3](#_Toc508110056)

[Supplementary Figure 1. Schematic of the custom-built fluorescence microscope. 3](#_Toc508110057)

[Supplementary Figure 2. Copy number of Mfd-YPet and mutants. 4](#_Toc508110058)

[Supplementary Figure 3. Representative fluorescence images 5](#_Toc508110059)

[Supplementary Figure 4. Mfd-YPet foci are lost when cells are treated with rifampicin. 6](#_Toc508110060)

[Supplementary Figure 5. Mfd-YPet molecules form more foci when cells are treated with CBR703. 7](#_Toc508110061)

[Supplementary Figure 6. Effect of CBR703 on RNAP residence time. 8](#_Toc508110062)

[Supplementary Figure 7. Simulation of *k*_eff_*τ*_tl_ plot using inputs obtained from global fitting. 9](#_Toc508110063)

[Supplementary Figure 8. Structural alignment of Mfd and the transcription initiation complex. 10](#_Toc508110064)

[Supplementary Tables 11](#_Toc508110065)

[Supplementary Table 1. Bacterial strains. 11](#_Toc508110066)

[Supplementary Table 2. Plasmids. 12](#_Toc508110067)

[Supplementary Table 3. Oligonucleotides used for λ Red recombination and cloning. 13](#_Toc508110068)

[Supplementary Table 4. Cumulative residence time distributions across multiple time-lapse times (*τ*_tl_) of Mfd-YPet in HH024 *mfd-ypet*. 14](#_Toc508110069)

[Supplementary Table 5. Cumulative residence time distributions across multiple time-lapse times 15](#_Toc508110070)

[Supplementary Table 6. Estimations of Mfd-YPet off-rates by linear fitting the *k*_eff_*τ*_tl_ plots 16](#_Toc508110071)

[Supplementary Table 7. Global fitting parameters and outputs 16](#_Toc508110072)

[Supplementary Notes 17](#_Toc508110073)

[1. Strain constructions 17](#_Toc508110074)

[2. Plasmid constructions 17](#_Toc508110075)

[3. Copy number of Mfd and mutants 18](#_Toc508110076)

[4. Dissociation kinetics of Mfd in *mfd-ypet* cells 18](#_Toc508110077)

[References 20](#_Toc508110078)

# Supplementary Figures


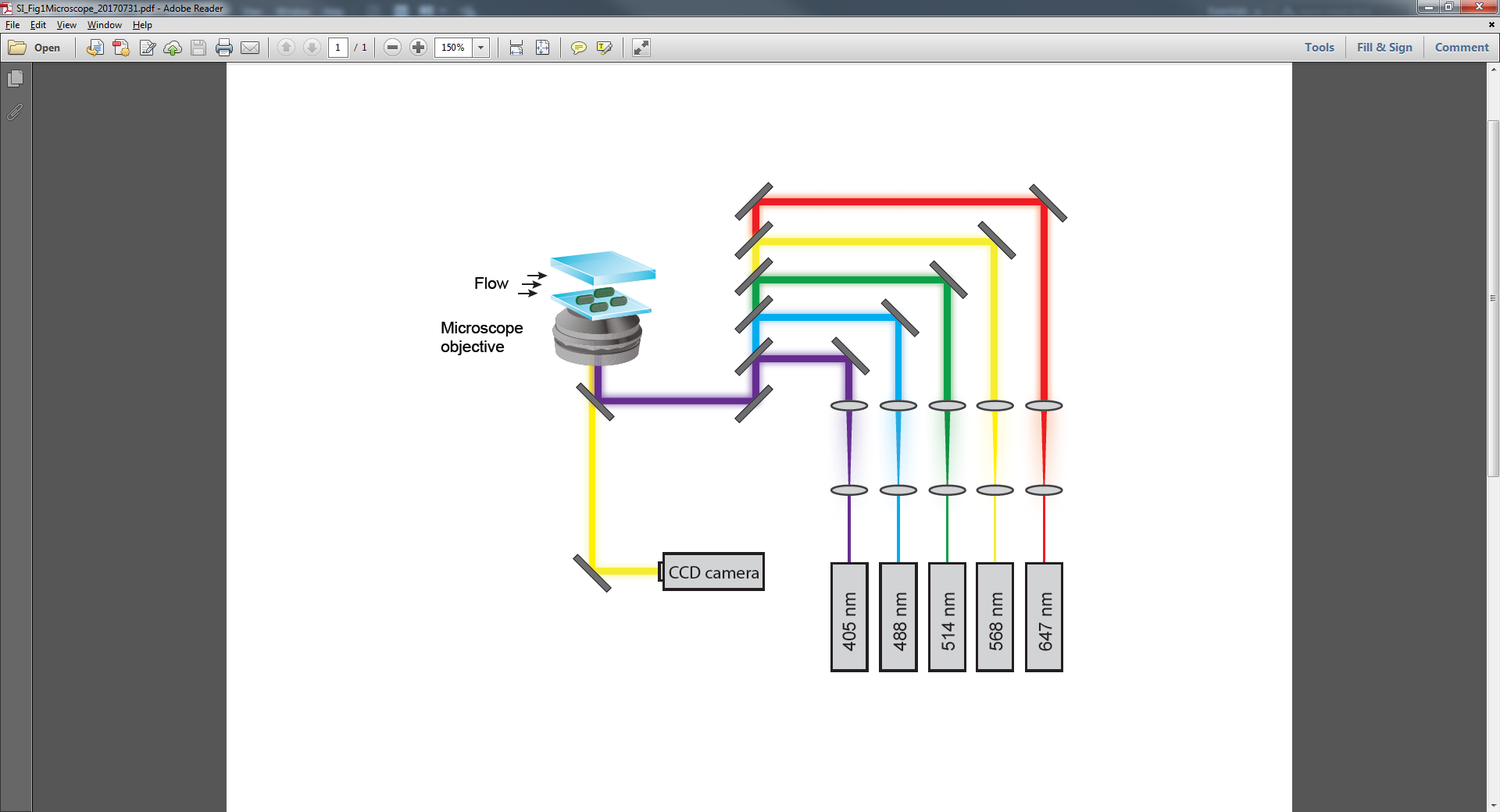


## **Supplementary Figure 1. Schematic of the custom-built fluorescence microscope.**

**
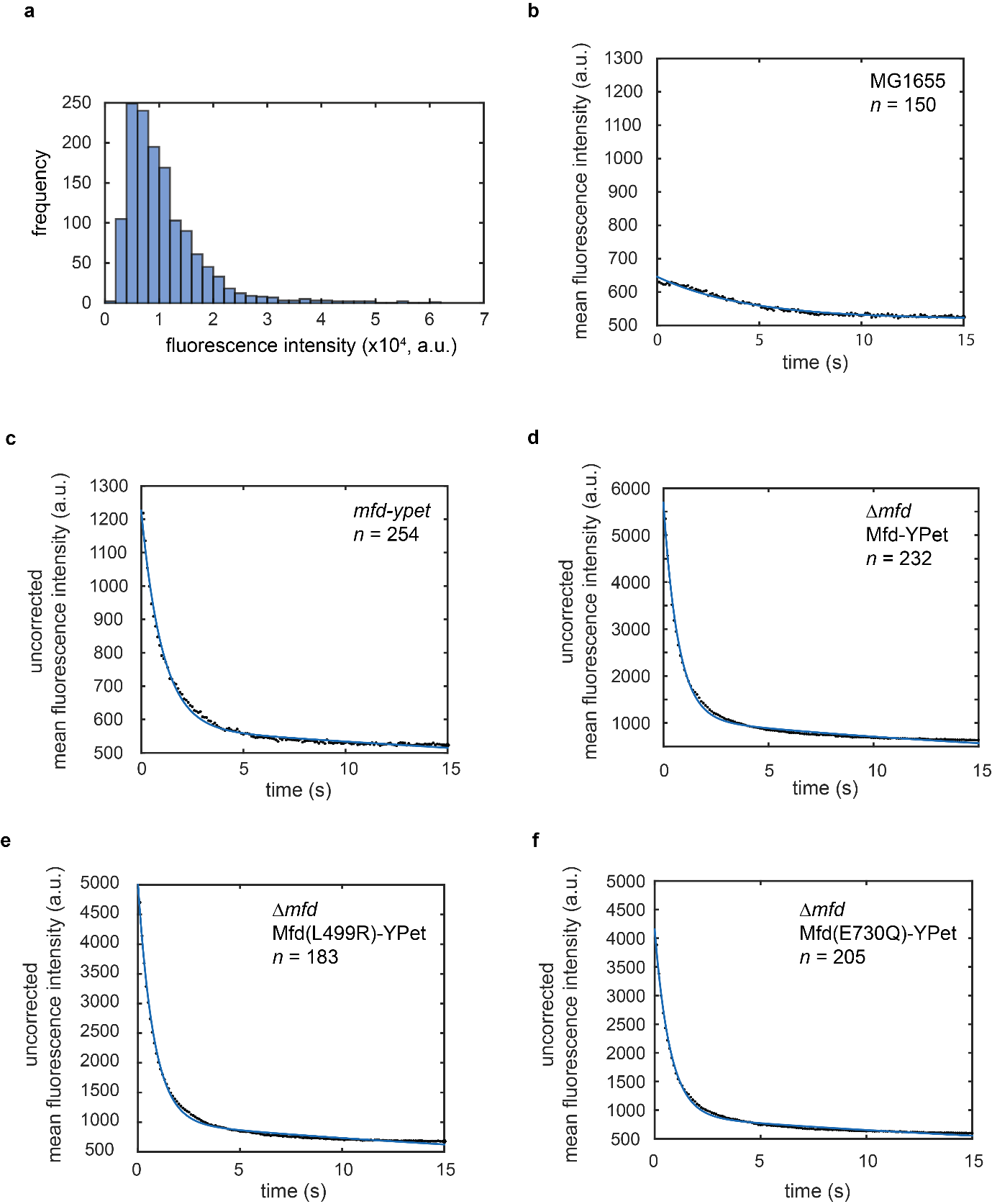
**

Supplementary Figure 2. Copy number of Mfd-YPet and mutants. (**a**) Histogram of fluorescence intensities of single molecules of Mfd-YPet in live cells (*n*_cells_ > 400). (**b**) Mean fluorescence intensity of wildtype MG1655 and uncorrected mean fluorescence intensity of (**c**) *mfd-ypet*, Δ*mfd* cells carrying plasmid-based (**d**) wildtype Mfd-YPet, (**e**) Mfd(L499R)-YPet or (**f**) Mfd(E730Q)-YPet with 514-nm illumination (10 fps). Blue lines are double-exponential fits to the averages of mean fluorescence intensity (closed circles). *n*, the number of cells. See Supplementary Note 3.

**
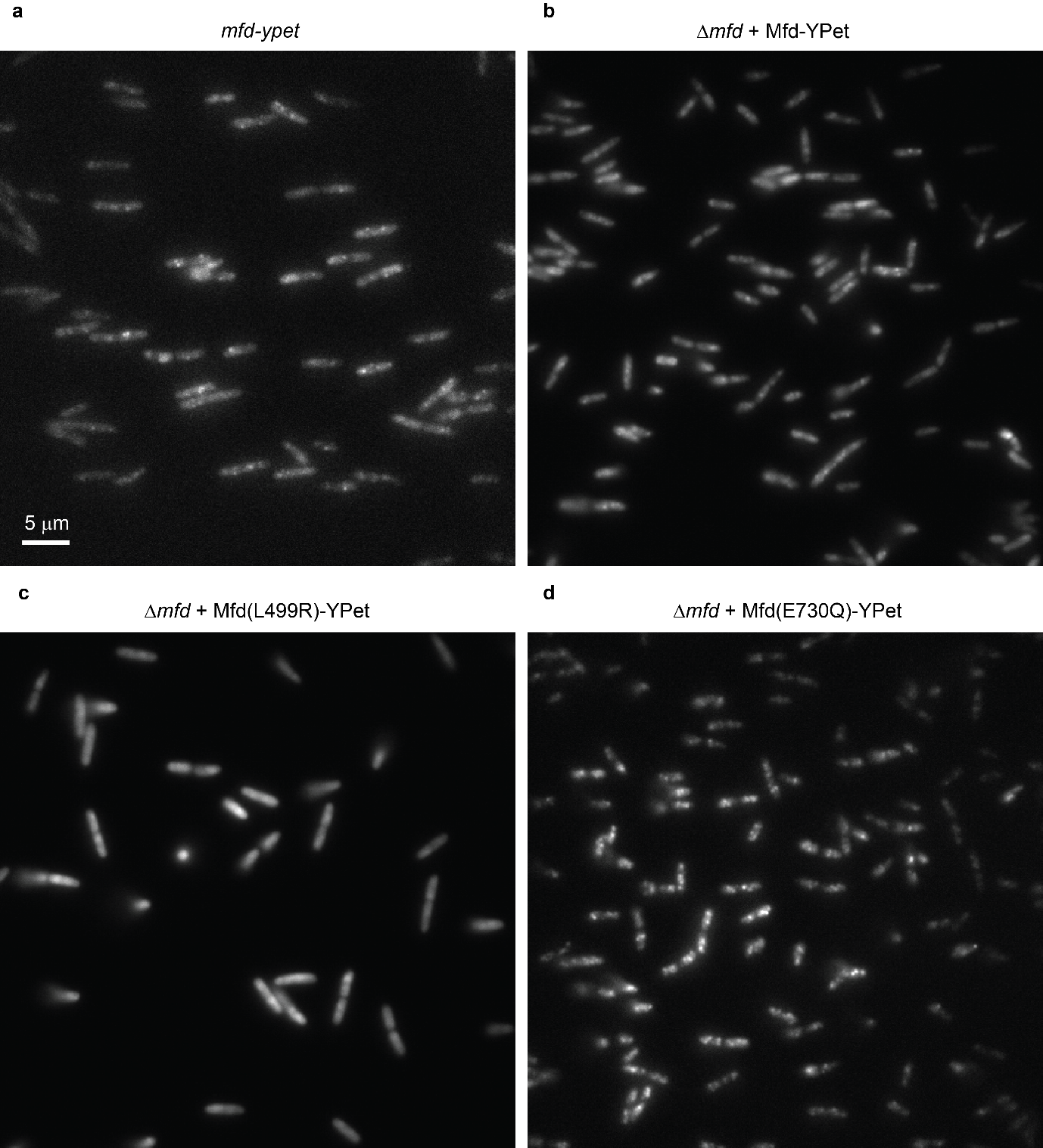
**

Supplementary Figure 3. Representative fluorescence images of (**a**) *mfd-ypet* cells and Δ*mfd* cells expressing plasmid-based (**b**) wildtype Mfd-YPet, (**c**) Mfd(L499R)-YPet or (**d**) Mfd(E730Q)-YPet. These images are average projections of the first 10 frames from rapid acquisitions (10 fps) acquired using 514-nm illumination. All fields of view are displayed on the same scale.


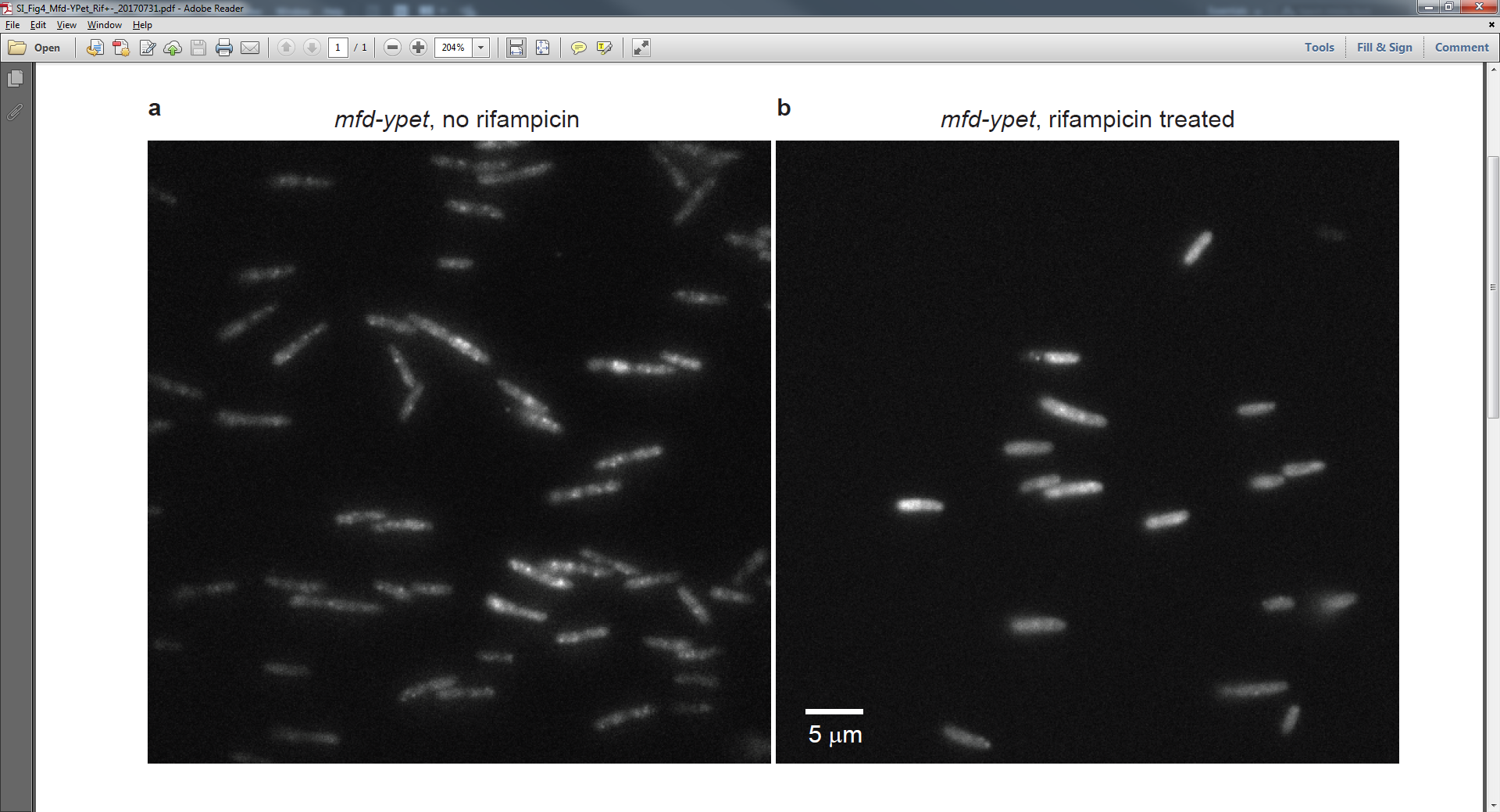


Supplementary Figure 4. Mfd-YPet foci are lost when cells are treated with rifampicin. Representative fluorescence images of *mfd-ypet* cells (**a**) in the absence of drug or (**b**) treated with rifampicin (50 µg per mL) and imaged after one hour. The images are average projections of the first 10 frames acquired using a rapid acquisition scheme (10 fps) with 514-nm illumination. All fields of view are displayed on the same scale.

**
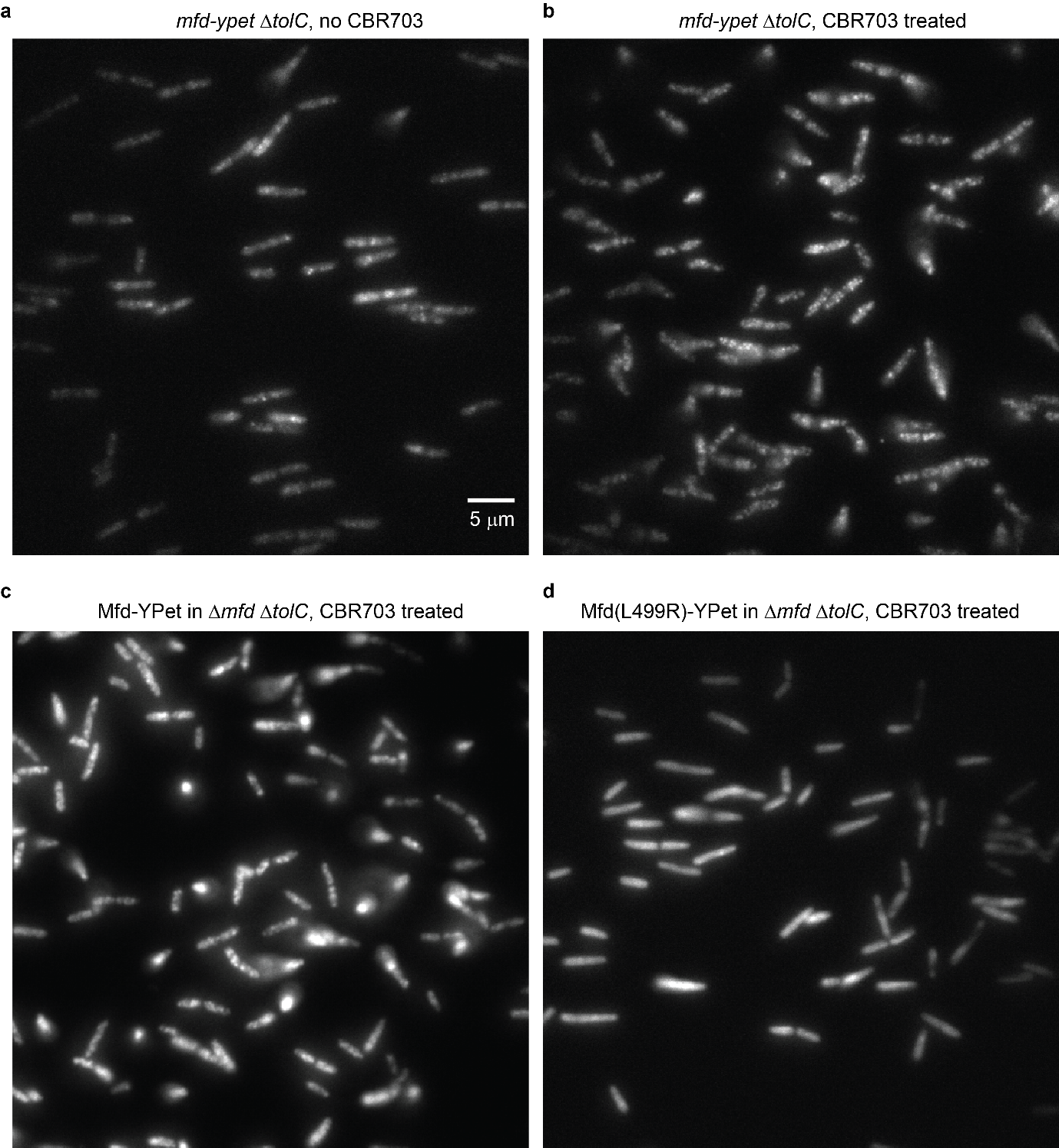
**

Supplementary Figure 5. Mfd-YPet molecules form more foci when cells are treated with CBR703. Representative fluorescence images of *mfd-ypet* Δ*tolC* cells imaged (**a**) in the absence of drug or (**b**) 30 minutes after treatment with CBR703 (75 µg per mL). The images are average projections of the first 10 frames acquired using a rapid acquisition scheme (10 fps) with 514-nm illumination. (**c-d**) Δ*mfd* Δ*tolC* cells expressing plasmid-based (**c**) Mfd-YPet and (**d**) Mfd(L499R)-YPet imaged after 30 minutes of treatment with CBR703 (75 µg per mL). All fields of view are displayed on the same scale.


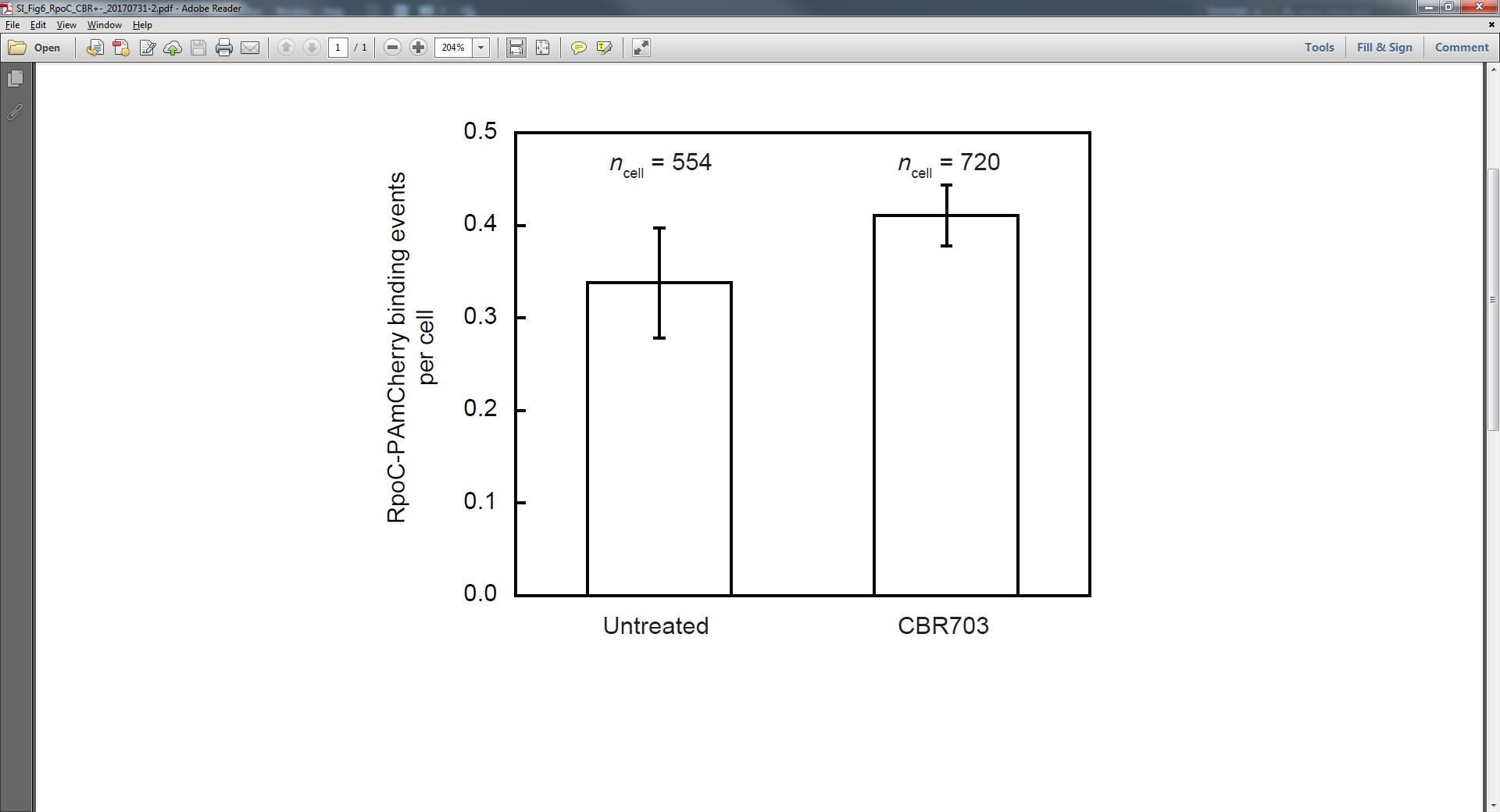


Supplementary Figure 6. Effect of CBR703 on RNAP residence time. *rpoC-PAmCherry* Δ*tolC* cells were imaged either in the absence of drug treatment or after a 30-minute treatment with CBR703 (75 µg per mL). Following pulse activation with the 405-nm laser, cells were imaged using the 568-nm read-out laser. Single RpoC-PAmCherry molecules that were present for at least two consecutive frames (200 ms) within two pixels were considered to represent bound RNAP. Bar plot represents number of bound RNAP foci detected per cell for untreated or CBR703 treated cells. Error bars represent standard deviations from three experiments.


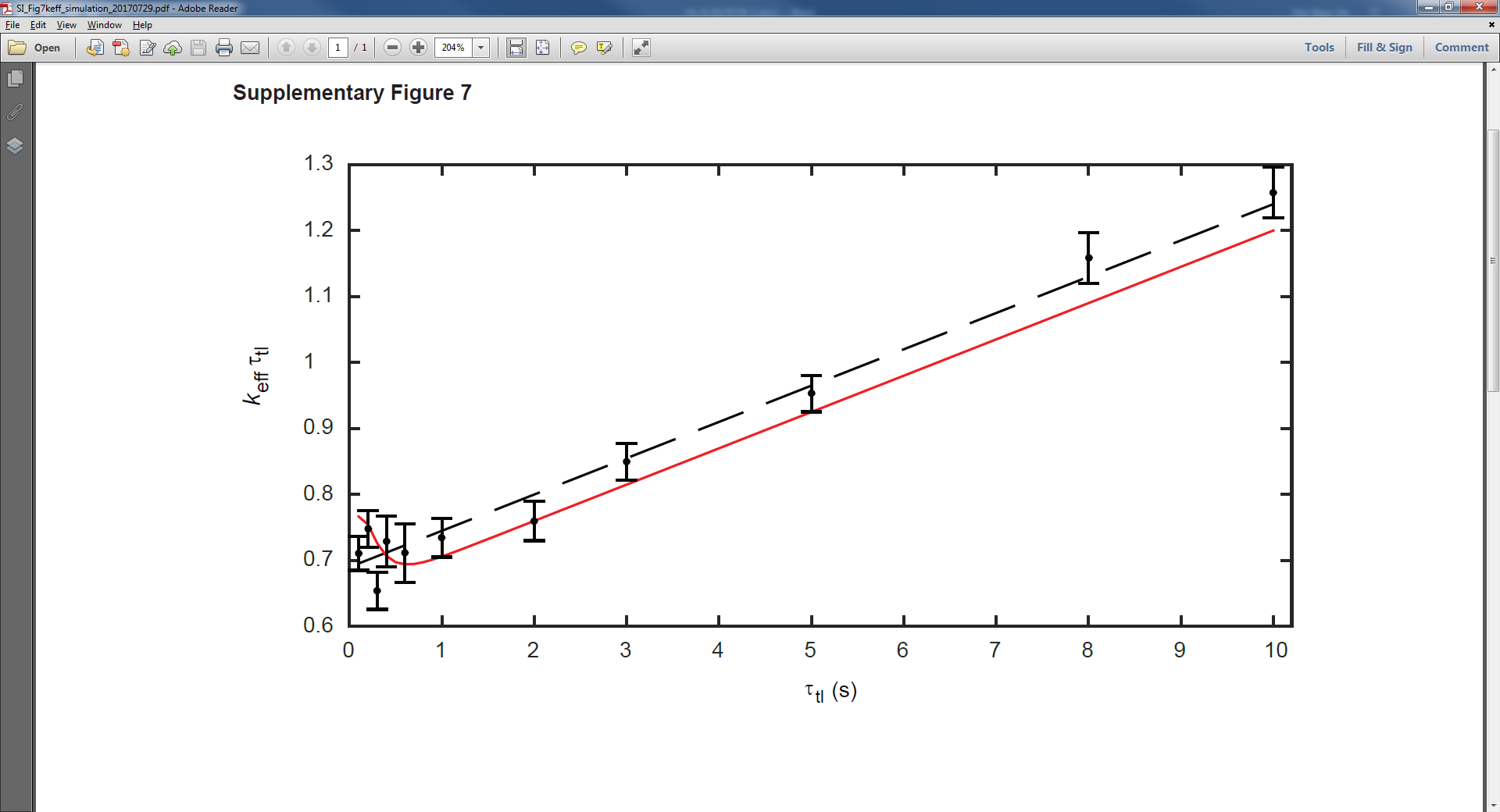


Supplementary Figure 7. Simulation of *k*_eff_*τ*_tl_ plot using inputs obtained from global fitting. *k*_eff_*τ*_tl_ plot for *mfd-ypet uvrA*^+^ (red curve in Fig. 4c, maintext) is represented as points. Error bars are standard deviations of the bootstrap distribution of *k*_eff_*τ*_tl_ for the corresponding time-lapse time. Black dashed line represents fit to equation 1 with *k*_off_ = 0.055 s^-1^. Red solid line represents fit to equation 3 with *k*_off1_ = 0.055 s^-1^, *B* = 0.58 and *k*_off2_ = 6 s^-1^. See Supplementary Table 7 and Supplementary Note 4.

**
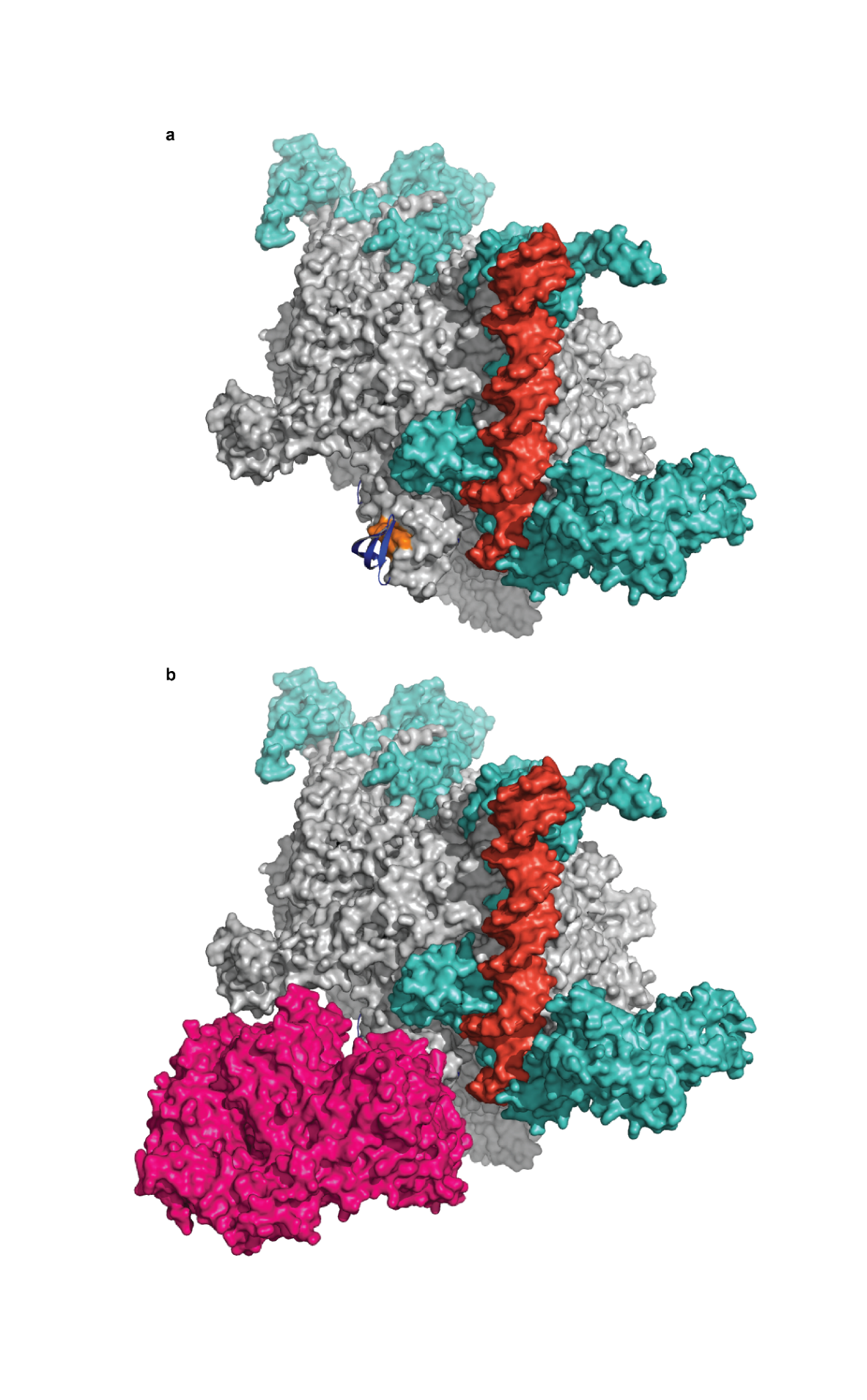
**

Supplementary Figure 8. Structural alignment of Mfd and the transcription initiation complex. (**a**) Structure of the *E. coli* transcription initiation complex, containing the core RNA polymerase (grey), σ^70^ (turquoise) and DNA (red) (PDB:4YLN^1^), was first aligned with the co-crystal structure of the *Thermus thermophilus* transcription-repair coupling factor (TRCF) RNA polymerase interacting domain (blue) and the *Thermus aquaticus* RNA polymerase β domain (PDB:3MLQ^2^). The residues of β subunit that are important for Mfd binding (I117 K118 E119) are highlighted in orange. (**b**) Further alignment with the Mfd apo crystal structure (hot pink, PDB:2EYQ^3^) was performed using homologous residues in the RNA polymerase interacting domains of *Ec*Mfd and *Thermus thermophilus* TRCF. Alignments were performed in PyMOL^4^. In this alignment, Mfd and σ^70^ can simultaneously bind the core enzyme.

# **Supplementary Tables**

Supplementary Table 1. Bacterial strains. All strains are in *E. coli* K-12 MG1655 background, with the exception of HH078 (BW25113). (*) Strains used for imaging. (**) Strains used for UV-survival assay. Primers are listed in Supplementary Table 3.

| **Strain** | **Relevant genotypes** | **Source/technique** |
| --- | --- | --- |
| MG1655 | F^-^, lambda^-^, rph-1 | Cox lab^5^ |
| HG015 | Δ*uvrA*::*kanR* | This study, λ Red recombination, primer6,7 |
| HH006 | Δ*mfd*::*kanR* | This study, λ Red recombination, primer2,3 |
| HH011 | Δ*mfd* | This study |
| HH016 | Δ*mfd* Δ*recA*::*kanR* | This study, λ Red recombination, primer4,5 |
| HH019 | *mfd-ypet kanR* | This study, λ Red recombination, primer1,3 |
| HH020 | Δ*recA*::*kanR* | This study, λ Red recombination, primer4,5 |
| HH021 (**) | Δ*recA* | This study |
| HH023 (**) | Δ*mfd* Δ*recA* | This study |
| HH024 (*) | *mfd-ypet* | This study |
| HH033 | *mfd-ypet* Δ*recA*::*kanR* | This study, λ Red recombination, primer 4,5 |
| HH034 (**) | *mfd-ypet* Δ*recA* | This study |
| HH036 (*) | *mfd-ypet* Δ*tolC* | This study |
| HH055 | *rpoC-PAmCherry1 amp* | Heilemann lab^6^ |
| HH078 | Δ*tolC*::*kanR* | Keio collection, CGSC #11430 |
| HH082 | *mfd-ypet* Δ*uvrA::kanR* | This study, P1 transduction |
| HH107 | *mfd-ypet* Δ*tolC*::*kanR* | This study, P1 transduction |
| HH109 | *rpoC-PAmCherry1 amp* Δ*tolC*::*kanR* | This study, P1 transduction |
| HH144 (*) | *mfd-ypet* Δ*uvrA* | This study |
| HH247 (*) | *rpoC-PAmCherry1 amp* Δ*tolC* | This study |
| HH366 (*) | Δ*mfd*/ pHH001 | This study, Supplementary Table 2, and Supplementary Note 2 |
| HH296 (*) | Δ*mfd*/ pHH002 | This study, Supplementary Table 2, and Supplementary Note 2 |
| HH393 (*) | Δ*mfd*/ pHH003 | This study, Supplementary Table 2, and Supplementary Note 2 |

## Supplementary Table 2. Plasmids.

| **Plasmid** | **Description** | **Source** |
| --- | --- | --- |
| pHH001 | Plasmid backbone carrying a pSC101 origin, spectinomycin marker and expresses Mfd-YPet under the native *mfd* promoter. | This study |
| pHH002 | Plasmid backbone carrying a pSC101 origin, spectinomycin marker and expresses Mfd (L499R)-YPet under the native *mfd* promoter. | This study |
| pHH003 | Plasmid backbone carrying a pSC101 origin, spectinomycin marker and expresses Mfd (E730Q)-YPet under the native *mfd* promoter. | This study |
| pJM1071 | Plasmid backbone carrying a pSC101 origin and spectinomycin marker. | Woodgate lab^7^ |

Supplementary Table 3. Oligonucleotides used for λ Red recombination and cloning. Homologies to the *E. coli* chromosome are underlined. The sequence encoding the 11-amino acid linker is bold.

| **Recombination primers** | **Sequence** |
| --- | --- |
| Primer 1  (Mfd_YPet_fw) | TCGAATGGGTACGCCAGTTTATGCGTGAACTGGAAGAGAACGCGATCGCT **TCGGCTGGCTCCGCTGCTGGTTCTGGCGAATTC**ATGTCTAAAGGTGAAGAATTATTCACTGGTG |
| Primer 2  (Mfd_del_507_fw) | ATATGCCCCCATATGTTGAGGCATATCCTAACGAGAATCTGACAACCGTT CCCTTTCGTCTTCAAGAATTC |
| Primer 3  (Mfd_del_507_rev) | GAATTTGTAAATGTTGCAGATGGGGGCGCAGAAACGCCCCCGATTTACCA GGCCACGATGCGTCCGGCGTA |
| Primer 4  (RecA_fw) | CAACAGAACATATTGACTATCCGGTATTACCCGGCATGACAGGAGTAAAA  CCCTTTCGTCTTCAAGAATTC |
| Primer 5  (RecA_rev) | AAAAAAGCAAAAGGGCCGCAGATGCGACCCTTGTGTATCAAACAAGACGA  GGCCACGATGCGTCCGGCGTA |
| Primer 6  (UvrA_del_507_fw) | CGGTA GCACC ATGCC ACCGG GCAAA AAAGC GTTTA ATCCG GGAAA GGTGA CCCTTTCGTCTTCAAGAATTC |
| Primer 7  (UvrA_del_507_rev) | GCTGG TGCAA CTCTG AAAGG AAAAG GCCGC TCAGA AAGCG GCCTT AACGA GGCCACGATGCGTCCGGCGTA |
| **Cloning primers** | **Sequence** |
| Primer 8  (pJM_Mfd_F) | CTGCATCAGGGCCCATTCAATTTGCTAAACCATGTCGAT |
| Primer 9  (pJM_Mfd_R) | GTCATGCTGAATTCGCCAGAACCAGCAGCGGAGCCAGCCGAAGCGATCGCGTTCTCTT |
| Primer 10  (pJM_YPet_F) | AACATATGGAATTCATGTCTAAAGGTGAAGAATTATTCACT |
| Primer 11  (pJM_YPet_R) | GCGGCCGCTCTAGATTAGAGCTCTTTGTACAATTCATTC |

Supplementary Table 4. Cumulative residence time distributions across multiple time-lapse times (*τ*_tl_) of Mfd-YPet in HH024 *mfd-ypet*.

| ***τ*_tl_ (s)** | | **0.1** | **0.2** | **0.3** | **0.4** | **0.6** | **1** | **2** | **3** | **5** | **8** | **10** |
| --- | --- | --- | --- | --- | --- | --- | --- | --- | --- | --- | --- | --- |
| **Number of consecutive frames** | **2** | 1974 | 1657 | 1689 | 929 | 734 | 1458 | 1620 | 2110 | 2482 | 1698 | 2336 |
|  | **3** | 841 | 676 | 733 | 387 | 311 | 621 | 670 | 811 | 878 | 524 | 620 |
|  | **4** | 462 | 356 | 426 | 215 | 170 | 334 | 353 | 392 | 385 | 168 | 232 |
|  | **5** | 280 | 190 | 285 | 123 | 111 | 194 | 212 | 217 | 221 | 75 | 92 |
|  | **6** | 182 | 134 | 198 | 88 | 72 | 114 | 129 | 126 | 120 | 31 | 37 |
|  | **7** | 130 | 96 | 143 | 64 | 44 | 78 | 83 | 82 | 62 | 14 | 20 |
|  | **8** | 88 | 64 | 96 | 41 | 30 | 47 | 51 | 55 | 34 | 10 | 11 |
|  | **9** | 68 | 45 | 64 | 29 | 22 | 34 | 31 | 32 | 20 | 7 | 7 |
|  | **10** | 49 | 31 | 45 | 21 | 11 | 24 | 22 | 25 | 11 | 4 | 3 |

Supplementary Table 5. Cumulative residence time distributions across multiple time-lapse times (*τ*_tl_) of Mfd-YPet in HH144 *mfd-ypet* Δ*uvrA*.

| ***τ*_tl_ (s)** | | **0.1** | **0.2** | **0.3** | **0.4** | **0.6** | **1** | **2** | **3** | **5** | **8** | **10** |
| --- | --- | --- | --- | --- | --- | --- | --- | --- | --- | --- | --- | --- |
| **Number of consecutive frames** | **2** | 1637 | 1447 | 1411 | 1426 | 643 | 1978 | 885 | 3187 | 1220 | 1677 | 5042 |
|  | **3** | 754 | 646 | 595 | 577 | 283 | 906 | 370 | 1388 | 507 | 575 | 1719 |
|  | **4** | 437 | 354 | 318 | 341 | 158 | 505 | 184 | 771 | 243 | 238 | 738 |
|  | **5** | 282 | 218 | 198 | 195 | 104 | 315 | 119 | 437 | 133 | 132 | 345 |
|  | **6** | 188 | 134 | 135 | 125 | 66 | 218 | 71 | 290 | 74 | 70 | 178 |
|  | **7** | 121 | 93 | 85 | 85 | 43 | 151 | 45 | 189 | 42 | 38 | 96 |
|  | **8** | 92 | 59 | 58 | 61 | 26 | 100 | 28 | 116 | 26 | 28 | 65 |
|  | **9** | 68 | 36 | 39 | 46 | 20 | 70 | 15 | 78 | 17 | 14 | 42 |
|  | **10** | 42 | 28 | 30 | 34 | 14 | 47 | 12 | 45 | 9 | 8 | 25 |

Supplementary Table 6. Estimations of Mfd-YPet off-rates by linear fitting the *k*_eff_*τ*_tl_ plots (see Supplementary Note 4).

| **Strain** | ***k*_b_ ± Error (s^-1^)** | ***k*_off_ ± Error (s^-1^)** | **Adjusted R-square** |
| --- | --- | --- | --- |
| HH024 *mfd-ypet* | 6.8 ± 0.1 | 0.056 ± 0.003 | 0.966 |
| HH144 *mfd-ypet* Δ*uvrA* | 6.5 ± 0.2 | 0.035 ± 0.004 | 0.897 |

Supplementary Table 7. Global fitting parameters and outputs (see Supplementary Note 4).

| **Fitting equation** | **Inputs** | | **Outputs** | | | |
| --- | --- | --- | --- | --- | --- | --- |
|  | **Fixed** | **Global** | ***k*_b_ ± Error**  **(s^-1^)** | ***k*_off1_ ± Error**  **(s^-1^)** | ***B*** | ***k*_off2_ ± Error**  **(s^-1^)** |
| **1** | *τ*_int_ | *k*_b_ > 0 s^-1^ | 6.9 ± 0.2 | 0.055 ± 0.005 | - | - |
|  | *τ*_tl_ | 10 s^-1^ ≥ *k*_off1_ ≥ 10^-3^ s^-1^ |  |  |  |  |
| **3** | *τ*_int_ | *k*_b_ > 0 s^-1^ | 6.8 ± 0.2 | 10^-3^ ± 0.07 | 0.1 ± 0.4 | 0.08 ± 0.04 |
|  |  | 10 s^-1^ ≥ *k*_off1_ ≥ 10^-3^ s^-1^ |  |  |  |  |
|  | *τ*_tl_ | 1 ≥ *B* ≥ 0 |  |  |  |  |
|  |  | 10 s^-1^ ≥ *k*_off2_ ≥ 10^-3^ s^-1^ |  |  |  |  |
| **3** | *τ*_int_ | *k*_b_ > 0 s^-1^ | 6.5 ± 0.2 | Fixed | 0.58 ± 0.09 | 6 ± 2 |
|  | *τ*_tl_ | 1 ≥ *B* ≥ 0 |  |  |  |  |
|  | *k*_off1_ = 0.055 s^-1^ | 10 s^-1^ ≥ *k*_off2_ ≥ 10^-3^ s^-1^ |  |  |  |  |

# Supplementary Notes

## Strain constructions

All strains (except HH078) are derivatives of *E. coli* K-12 MG1655 ^5^, and were created either by λ Red recombination^8^, P1 transduction^9^ or obtained as listed in Supplementary Table 1.

- 1. **HH019 *mfd-ypet* *kanR* and HH024 *mfd-ypet***

The strain HH019 *mfd-ypet kanR* expresses the C-terminal Mfd-YPet fusion protein with an 11-amino-acid linker (SAGSAAGSGEF^10^) from the native chromosomal locus of *mfd*. This strain was constructed from MG1655 using λ Red recombination (standard protocols^8^). Primers for recombineering are indicated in Supplementary Table 3. After recombination, colonies were selected for the presence of the KanR marker, and the genetic fusion *mfd-ypet* was verified with DNA sequencing. The kanamycin cassette in HH019 was removed to create HH024 *mfd-ypet*, containing a mutant FRT site.

- 1. **HH006 Δ*mfd*::*kanR*, HG015 Δ*uvrA*::*kanR* and HH011 Δ*mfd***

Similarly, HH006 Δ*mfd*::*kanR* and HG015 Δ*uvrA*::*kanR* were constructed *de novo* from MG1655 using λ Red recombination. *mfd* and *uvrA* genes were replaced with the mutant FRT-kanamycin resistance-FRT cassette amplified from pEAW507^11^. From HH006, the kanamycin resistance cassette was removed to create HH011 Δ*mfd.*

- 1. **HH020 Δ*recA*::*kanR,* HH016 Δ*mfd* Δ*recA*::*kanR* and HH033 *mfd-ypet* Δ*recA*::*kanR***

HH020, HH016 and HH033 were created from MG1655, HH011 and HH024 cells respectively by replacing *recA* with the mutant FRT-kanamycin resistance-FRT cassette amplified from pEAW507^11^. The kanamycin resistance cassettes were removed to create HH021, HH023 and HH034; these strains were used in UV-survival assays.

- 1. **HH107 *mfd-ypet* Δ*tolC*::*kanR* and HH109 *rpoC-PAmCherry1 amp* Δ*tolC*::*kanR***

Δ*tolC*::*kan*R was transduced into HH024 and HH055, using P1 transduction^9^, to create HH107 and HH109 respectively. The kanamycin resistance cassettes were removed to create HH036 and HH247.

- 1. **HH082 *mfd-ypet* Δ*uvrA*::*kanR* and HH144 *mfd-ypet* Δ*uvrA***

Δ*uvrA*::*kan*R was transduced into HH024, using P1 transduction^9^, to create HH082. The kanamycin resistance cassette was removed to create HH144.

## Plasmid constructions

pHH001 and pHH002 carry the *mfd-ypet* and *mfd(L499R)-ypet* allele respectively under the native *mfd* promoter. These plasmids were constructed by restriction cloning followed by three-fragment ligation into pJM1071 (a gift from the Woodgate laboratory) between the *Apa*I and *Xba*I sites. This vector is a low-copy number plasmid with a pSC101 origin and carries a spectinomycin marker. Wildtype *mfd* and *mfd(L499R)* genes under the native *mfd* promoter (106 nucleotides directly upstream of *mfd* gene) were amplified from the MG1655 chromosome and pMfd2-LR499^3^ respectively. PCR products were purified and digested with *Apa*I and *EcoR*I. Simultaneously, the *ypet* gene was digested with *EcoR*I and *Xba*I. Three-fragment ligation was carried out at 16 °C overnight with T4 DNA ligase. The ligation mixture was subsequently transformed into DH5α. Resultant colonies were screened with colony PCR. Finally, plasmids were isolated and verified to possess the correct sequences. Using the same protocol, pHH003 carry the *mfd(E730Q)-ypet* allele was created by sub-cloning *mfd(E730)* gene (gBlocks, IDT, US) into pHH001 between *Xho*I and *AsiS*I sites.

## Copy number of Mfd and mutants

Copy numbers of Mfd and mutants were determined by dividing the integrated cellular fluorescence with the mean fluorescence intensity of single molecules, essentially as described earlier^12^. Single molecules were detected as described in Methods (main text) and fitted to 2-D Gaussian functions. The volume of the 2-D Gaussian function represents the fluorescence intensity of single molecule Mfd-YPet and follows normal distribution (Supplementary Fig. 2a).

The integrated cellular fluorescence was determined by multiplying the mean fluorescence intensity with the mean cell area (372 pixel^2^). The mean fluorescence intensity was obtained as follows:

- The photobleaching curves of *mfd-ypet* (HH024, *n*_cell_ = 254), Δ*mfd* cells expressing Mfd-YPet (HH366, *n*_cell_ = 232), Mfd(L499R)-YPet (HH296, *n*_cell_ = 183) and Mfd(E730Q)-YPet (HH393, *n*_cell_ = 205) (Supplementary Fig. 2c-f, closed circles) were obtained from rapid acquisitions (10 fps) with 514-nm illumination. These data were then fit to a double-exponential decay model (*f(x) = ae^bx^+ce^dx^, b* < 0, *d* < 0; blue lines) to obtain the uncorrected mean fluorescence intensities (blue line y-intercepts).
- The auto-fluorescence of MG1655 and the fluorescence of the coverslip were obtained from photobleaching curves of MG1655 (Supplementary Fig. 2b) in a similar manner.

The mean cellular fluorescence intensities were obtained by subtracting the auto-fluorescence of MG1655 from the uncorrected mean fluorescence intensities.

## Dissociation kinetics of Mfd in *mfd-ypet* cells

We used interval imaging to distinguish photobleaching from dissociation events essentially as described previously^13^. A cumulative residence time distribution as a function of frame-time was compiled for each *τ*_tl_ from nine independent experiments (Supplementary Table 4). Frame time was converted to real time using the formula *t* = *(n -1)τ_tl_* with *n* being the number of frames a binding event can be observed (Fig. 4b). The effective off-rate constant *k*_eff_, representing a mixture of the off-rate constant (*k*_off_) and the photobleaching constant (*k*_b_), was obtained by fitting the cumulative residence time distribution to a single exponential decay model as in equation 1:

$f_{1}\left( t \right)=A\exp\left( -k_{\mathrm{eff}}t \right)=A\exp\left( -\left( k_{b}\frac{\tau_{\mathrm{int}}}{\tau_{\mathrm{tl}}}+k_{\mathrm{off}} \right)t \right)$ (1)

where *A* is the number of molecules, *t* is real time in seconds^13^.

To determine uncertainties in *k*_eff_*τ*_tl_, fitting was performed a thousand times on cumulative residence time distribution derived from randomly selecting 80% of the binding events (bootstrapping and fitting performed with custom MATLAB code, MathWorks).

Equation (1) can be rearranged:

$k_{eff}\tau_{tl}=k_{b}\tau_{int}+k_{off}\tau_{tl}$ (2)

By plotting *k*_eff_*τ*_tl_ as a function of *τ*_tl_ (Fig. 4c), *k*_off_ and *k*_b_*τ*_int_ were derived from the slope (*k*_off_ = 0.056 ± 0.003 s^-1^) and the *y*-intercept (*k*_b_*τ*_int_ = 0.68 ± 0.01) respectively, using linear least squares minimization (OriginPro 2015, OriginLab, MA, US).

Alternatively, similar outputs were obtained by global fitting cumulative residence time distribution across *τ*_tl_ using equation (1) as the fitting model (Origin Pro 2015, OriginLab, MA, US) (Supplementary Table 7). Minimization was performed using the Levenberg Marquardt algorithm and was stopped when a tolerance value smaller than 10^-9^ was met.

The sub-second fraction of *k*_eff_*τ*_tl_ deviates from the linear fit, indicating the presence of transient interactions. Assuming that these transient interactions arise out of a second population with a faster off-rate constant (*k*_off2_; *k*_off2_ > *k*_off1_) (Supplementary Fig. 7)^13^. The model describing the dissociation of Mfd then becomes:

$f_{2}\left( t \right)=A \left( B\exp\left( -\left( k_{b}\frac{\tau_{\mathrm{int}}}{\tau_{\mathrm{tl}}}+k_{off1} \right)t \right)+\left( 1-B \right)\exp\left( -\left( k_{b}\frac{\tau_{\mathrm{int}}}{\tau_{\mathrm{tl}}}+k_{off2} \right)t \right) \right)$ (3)

Where *A* is the number of molecules, *t* is real time in seconds and *B* represents the percentage of Mfd molecules exhibiting off-rate constant *k*_off1­_. We attempted to fit the cumulative residence time distributions to equation (3), using global fitting as described earlier (Supplementary Table 7). When *k*_off1_ was constrained to be 0.055 s^-1^, the resulting reduced *X*^2^ value of this fitting is smaller than that of global fitting to equation (1) (1970 < 2251), indicating a better fit was obtained using equation (3). This is simulated in Supplementary Fig. 7 (solid red line). The presence of a second population (42%) with a faster off-rate constant (6 ± 2 s^-1^) may represent Mfd in search for substrates or non-specific binding of Mfd with DNA.

# References

1 Zuo, Y. & Steitz, T. A. Crystal structures of the E. coli transcription initiation complexes with a complete bubble. *Molecular cell* **58**, 534-540, doi:10.1016/j.molcel.2015.03.010 (2015).

2 Westblade, L. F. *et al.* Structural basis for the bacterial transcription-repair coupling factor/RNA polymerase interaction. *Nucleic Acids Res* **38**, 8357-8369, doi:10.1093/nar/gkq692 (2010).

3 Deaconescu, A. M. *et al.* Structural Basis for Bacterial Transcription-Coupled DNA Repair. *Cell* **124**, 507-520 (2006).

4 Schrodinger, LLC. *The PyMOL Molecular Graphics System, Version 1.8* (2015).

5 Blattner, F. R. *et al.* The complete genome sequence of Escherichia coli K-12. *Science (New York, N.Y.)* **277**, 1453-1462 (1997).

6 Endesfelder, U. *et al.* Multiscale spatial organization of RNA polymerase in Escherichia coli. *Biophysical journal* **105**, 172-181, doi:10.1016/j.bpj.2013.05.048 (2013).

7 Churchward, G., Belin, D. & Nagamine, Y. A pSC101-derived plasmid which shows no sequence homology to other commonly used cloning vectors. *Gene* **31**, 165-171 (1984).

8 Datsenko, K. A. & Wanner, B. L. One-step inactivation of chromosomal genes in Escherichia coli K-12 using PCR products. *Proceedings of the National Academy of Sciences of the United States of America* **97**, 6640-6645, doi:10.1073/pnas.120163297 (2000).

9 Miller, J. H. *Experiments in Molecular Genetics*. (Cold Spring Harbor Laboratory Press, 1972).

10 Reyes-Lamothe, R., Sherratt, D. J. & Leake, M. C. Stoichiometry and architecture of active DNA replication machinery in Escherichia coli. *Science (New York, N.Y.)* **328**, 498-501, doi:10.1126/science.1185757 (2010).

11 Kim, T. *et al.* Directed Evolution of RecA Variants with Enhanced Capacity for Conjugational Recombination. *PLoS Genet* **11**, e1005278, doi:10.1371/journal.pgen.1005278 (2015).

12 Robinson, A. *et al.* Regulation of Mutagenic DNA Polymerase V Activation in Space and Time. *PLoS Genet* **11**, e1005482, doi:10.1371/journal.pgen.1005482 (2015).

13 Gebhardt, J. C. *et al.* Single-molecule imaging of transcription factor binding to DNA in live mammalian cells. *Nature methods* **10**, 421-426, doi:10.1038/nmeth.2411 (2013).
