# Supplementary material for: The transcription-repair coupling factor Mfd associates with RNA polymerase in the absence of exogenous damage
Source: Nat Commun. 2018 Apr 20;9:1570. doi: 10.1038/s41467-018-03790-z (PMC5910403; doi:10.1038/s41467-018-03790-z)
Supplement: Supplementary file 2 — Description of Additional Supplementary Files [file 41467_2018_3790_MOESM2_ESM.pdf]

Descriptions of Additional Files

File Name: Supplementary Movie 1
Descriptions: Mfd-YPet associates stably with the nucleoid. Rapid acquisition imaging (collected at 10 fps) of mfd-ypet cells (HH024) with 514-nm illumination. Scale bar represents 2 µm.

File Name: Supplementary Movie 2
Descriptions: Mfd(L499R)-YPet exhibits transient binding. Rapid acquisition imaging (collected at 10 fps) of Δmfd cells expressing Mfd(L499R)-YPet (HH296) with 514-nm illumination. Scale bar represents 2 µm.
